# Supplementary material for: Causal Relationship between Aging and Anorexia Nervosa: A White-Matter-Microstructure-Mediated Mendelian Randomization Analysis
Source: Biomedicines. 2024 Aug 16;12(8):1874. doi: 10.3390/biomedicines12081874 (PMC11351342; doi:10.3390/biomedicines12081874)
Supplement: Supplementary file 1 [file biomedicines-12-01874-s001.zip › Supplementary Figure/Figure S4/Singe SNP plot of MR analysis for TL to ubm-b-1475.pdf]

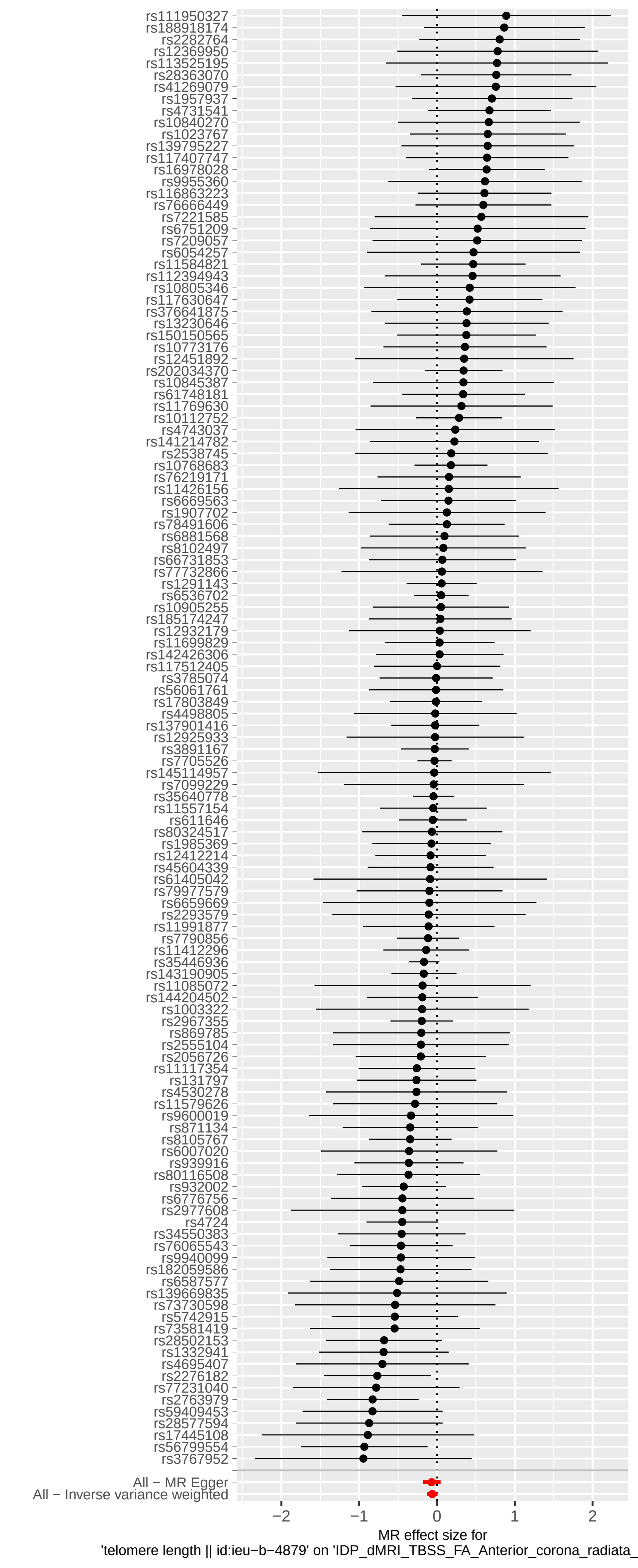
rs111950327
rs188918174
rs2282764
rs12369950
rs113525195
rs28363070
rs41269079
rs1957937
rs4731541
rs10840270
rs1023767
rs139795227
rs117407747
rs16978028
rs9955360
rs116863223
rs76666449
rs7221585
rs6751209
rs7209057
rs6054257
rs11584821
rs112394943
rs10805346
rs117630647
rs376641875
rs13230646
rs150150565
rs10773176
rs12451892
rs202034370
rs10845387
rs61748181
rs11769630
rs10112752
rs4743037
rs141214782
rs2538745
rs10768683
rs76219171
rs11426156
rs6669563
rs1907702
rs78491606
rs6881568
rs8102497
rs66731853
rs77732866
rs1291143
rs6536702
rs10905255
rs185174247
rs12932179
rs11699829
rs142426306
rs117512405
rs3785074
rs56061761
rs17803849
rs4498805
rs137901416
rs12925933
rs3891167
rs7705526
rs145114957
rs7099229
rs35640778
rs11557154
rs611646
rs80324517
rs1985369
rs12412214
rs45604339
rs61405042
rs79977579
rs6659669
rs2293579
rs11991877
rs7790856
rs11412296
rs35446936
rs143190905
rs11085072
rs144204502
rs1003322
rs2967355
rs869785
rs2555104
rs2056726
rs11117354
rs131797
rs4530278
rs11579626
rs9600019
rs871134
rs8105767
rs6007020
rs939916
rs80116508
rs932002
rs6776756
rs2977608
rs4724
rs34550383
rs76065543
rs9940099
rs182059586
rs6587577
rs139669835
rs73730598
rs5742915
rs73581419
rs28502153
rs1332941
rs4695407
rs2276182
rs77231040
rs2763979
rs59409453
rs28577594
rs17445108
rs56799554
rs3767952
All − MR Egger
All − Inverse variance weighted
−2
−1
0
1
2
MR effect size for
'telomere length || id:ieu−b−4879' on 'IDP_dMRI_TBSS_FA_Anterior_corona_radiata_
